# New Appliances and Things Medical

**Published:** 1902-04-05

**Authors:** 


### New Appliances and Things Medical.

[We shall be glad to receive, at our Office, 28 & 29 Southampton Street, Strand, London, W.C., from the manufacturers, specimens of all new preparations and appliances which may be brought out from time to time.]

### WEIGHT CHARTS.

(S. Maw, Son and Sons, Aldersgate Street, E.C.)

Mr. Kelf, of the St. Saviour's Infirmary, East Dulwich, has devised a series of charts on which to record the weights of patients, a detail which is likely to become one of growing importance as more attention is paid to questions of nutrition. These charts were designed for use in the Southwark Infirmary, where all the phthisical patients are regularly weighed once a week and the weight recorded. There can be no doubt about the advantage of "charting" all facts which can be recorded by the graphic method, which of course is eminently the case in matters of weight. The charts are prepared in three forms, No 1 going by half-pounds from one pound to 3 stones, No. 2 in pounds from 3 to 9 stones, and No. 3 in the same way from 8 to 14 stones, each chart running for 40 weeks. They are likely to be very useful.

